# Silk Fibroin-Based Biomaterials for Tissue Engineering Applications

**DOI:** 10.3390/molecules27092757

**Published:** 2022-04-25

**Authors:** Guangfei Li, Shan Sun

**Affiliations:** ENT Institute and Otorhinolaryngology Department of Eye & ENT Hospital, State Key Laboratory of Medical Neurobiology and MOE Frontiers Center for Brain Science, Fudan University, Shanghai 200031, China; 15216138952@163.com

**Keywords:** tissue engineering, silk fibroin, biomaterial

## Abstract

Tissue engineering (TE) involves the combination of cells with scaffolding materials and appropriate growth factors in order to regenerate or replace damaged and degenerated tissues and organs. The scaffold materials serve as templates for tissue formation and play a vital role in TE. Among scaffold materials, silk fibroin (SF), a naturally occurring protein, has attracted great attention in TE applications due to its excellent mechanical properties, biodegradability, biocompatibility, and bio-absorbability. SF is usually dissolved in an aqueous solution and can be easily reconstituted into different forms, including films, mats, hydrogels, and sponges, through various fabrication techniques, including spin coating, electrospinning, freeze drying, and supercritical CO_2_-assisted drying. Furthermore, to facilitate the fabrication of more complex SF-based scaffolds, high-precision techniques such as micro-patterning and bio-printing have been explored in recent years. These processes contribute to the diversity of surface area, mean pore size, porosity, and mechanical properties of different silk fibroin scaffolds and can be used in various TE applications to provide appropriate morphological and mechanical properties. This review introduces the physicochemical and mechanical properties of SF and looks into a range of SF-based scaffolds that have recently been developed. The typical applications of SF-based scaffolds for TE of bone, cartilage, teeth and mandible tissue, cartilage, skeletal muscle, and vascular tissue are highlighted and discussed followed by a discussion of issues to be addressed in future studies.

## 1. Introduction

One of the most serious problems in human healthcare is the repair of damaged and degenerated tissues as well as failed organs, and these issues present many challenges to modern medicine. Tissues such as bone, tendons, cartilage, peripheral nerves, and skin are prone to destruction via trauma, aging, and diseases such as osteoarthritis, Parkinson’s disease, and Alzheimer’s disease, and such tissue loss negatively affects the quality of life of millions of people around the world and puts enormous pressure on the global healthcare system [1]. In general, autografting and allografting are common clinical techniques to replace damaged tissue, but they are limited by various factors such as a lack of tissue in healthy areas that can be removed from the patient and a lack of suitable donors [2]. The success rates of allografts can be low because donor tissue from others can lead to an immune response, and in the case of extensive damage and large surface areas, it is hard to source suitable material in time leading to low success rates [2]. It is for these reasons that tissue engineering (TE) has attracted increasing attention as an alternative method for producing patient-specific tissues for repair and replacement applications.

TE combines several principles and methods and seeks to restore, maintain, and improve tissue functions in order to regenerate damaged tissues and organs, and TE relies on the use of diverse biocompatible materials that can be seeded with cells and that contain various supportive moieties such as growth factors [3]. The following properties should be considered when designing a biomaterial for TE applications regardless of the tissue type: (1) excellent biocompatibility in vivo; (2) optimized physical properties, especially mechanical properties; (3) ability to provide topographic and morphological cues; (4) biodegradability with safe by-products; and (5) the ability to control diffusion [4]. The extracellular matrix (ECM) secreted from tissues or organs is an excellent natural option as a scaffold material for TE because it exists in a state of “dynamic reciprocity” with resident cells [5]. Therefore, ECM components such as collagen [6], fibronectin [7], and glycosaminoglycan [8] have been widely used as natural scaffold materials to support tissue regeneration applications. In addition, other natural polymers such as alginate [9], cellulose [10], and chitosan [11] have been used in TE applications. Although these natural materials have demonstrated excellent results, they also have several drawbacks, including high costs, poor mechanical properties, and large batch-to-batch variation, thus making them difficult to use in clinical applications. On the other hand, synthetic materials such as polylactic acid (PLA), polyurethane (PU), poly(lactide-co-glycolide) (PLGA), and polycaprolactones (PCL) are widely used in TE because of their excellent structural properties and biocompatibility and efficient biodegradation [12]. However, many degradation products of these polymers consist of acidic compounds that are harmful to the body and can cause undesired immune responses. Because most of the natural and synthetic polymeric scaffolds have inherent limitations, finding a biomaterial that combines the benefits of natural and synthetic polymeric materials has become the focus of much research in recent decades [13].

**Figure 1:**
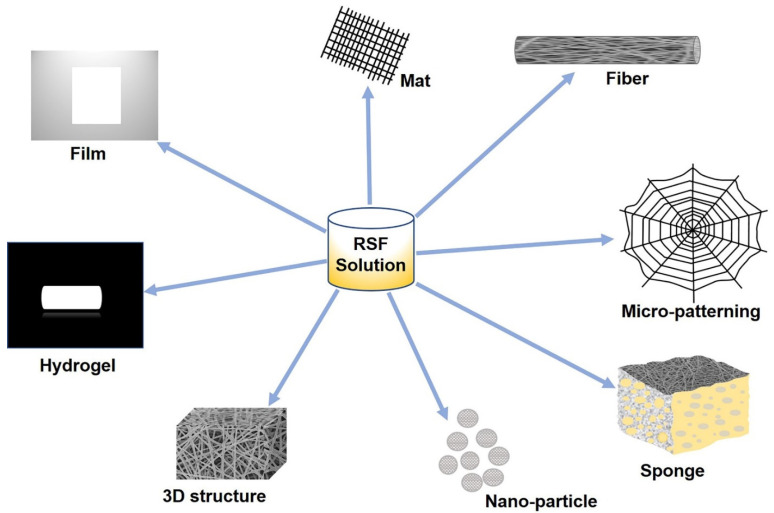
SF-based scaffolds with different morphologies—film, mat, fiber, micro-patterning, sponge, nano-particle, 3D structure, and hydrogel.

**Figure 2:**
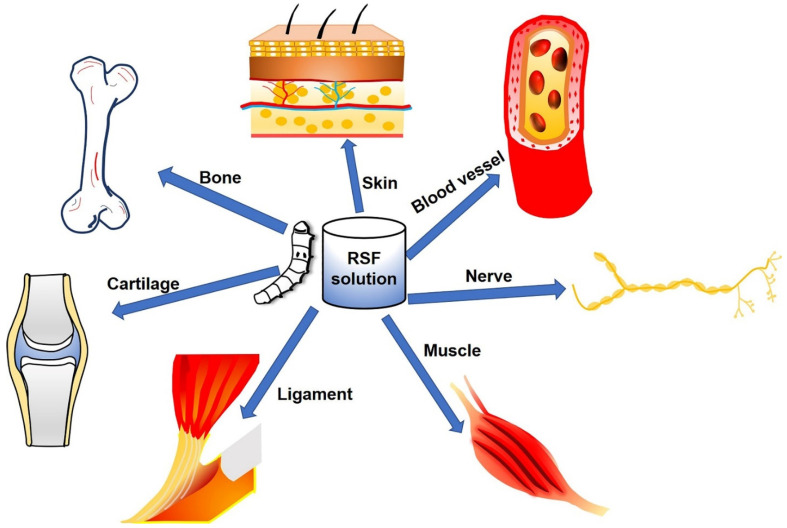
SF-based biomaterials applied in TE, including hard tissues (bone and cartilage) and soft tissues (ligaments, skin, blood vessels, nerves, and muscles).

Recent studies have explored the possibilities of silkworm silk as an excellent biomaterial for TE scaffolds that can potentially overcome many of the limitations of existing materials. Silk fibroin (SF), which is extracted from silkworm silk, is a unique natural protein that has been used as a potential biopolymer for TE based on the following physiochemical properties: (1) biocompatibility, (2) tunable biodegradation, (3) minimal immunogenicity, (4) adaptability to various formats (three-dimensional (3D) scaffolds, thin films, nanofibers, microspheres, nanoparticles, hydrogels, etc.) (Figure 1), (5) excellent mechanical strength, (6) easy accessibility, (7) cost effectiveness, and (8) environmentally friendly processing techniques [14,15,16]. SF can also be combined with other polymers to form SF-based composite scaffolds that can synergistically promote cellular behaviors such as differentiation, proliferation, and attachment [17,18], and it is possible to fabricate SF-based biomaterials into various material formats such as solutions, powders, nanoparticles [19], fibers, mats [20], films [21], hydrogels [22], sponges, and 3D structures [23]. In this review, we introduce SF’s properties and its diverse morphologies according to various fabrication methods and applications with an emphasis on the regeneration of hard tissues (bones, teeth, osteochondral, and cartilage) and soft tissues (skeletal muscle and vascular tissues) (Figure 2) (Table 1).

We searched the Web of Science and PubMed databases for articles using the search terms “spider silk”, “silk fibroin”, “biomaterials”, “composition”, “mechanical properties”, “tissue engineering”, “bone”, “cartilage”, “teeth”, and “mandible tissue”, “skeletal muscle”, and “vascular tissue” and compiled an across-species database. We obtained the full text of all articles that reported the tensile mechanical properties and/or the amino acid composition of SF and all articles that reported the diversity of SF forms and uses in various TE applications.

## 2. The Properties of SF

**Figure 3:**
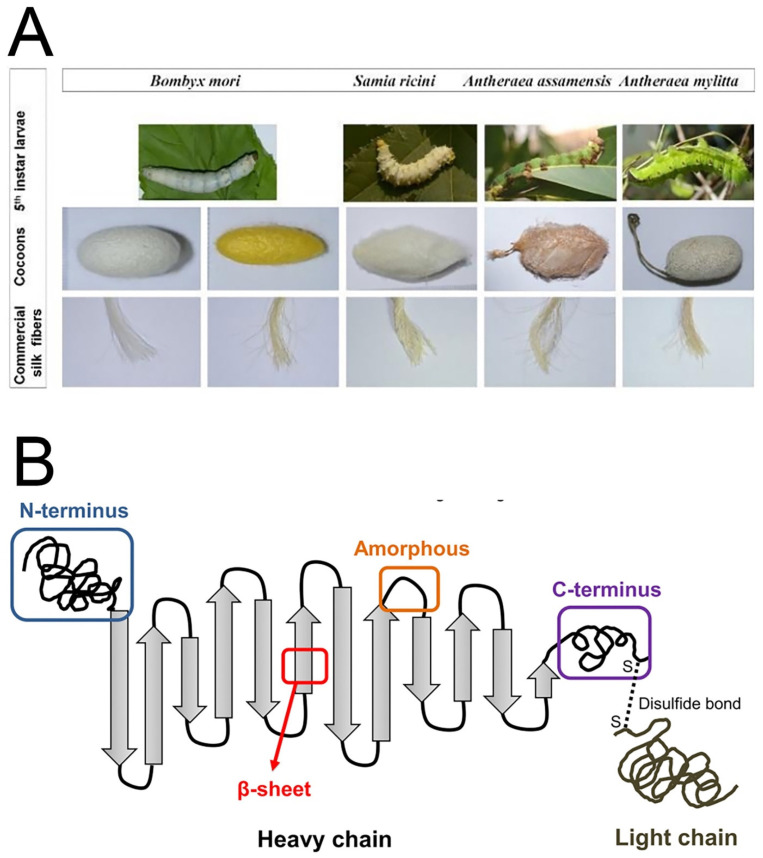
Schematic diagram of the silk source and structure. (**A**) Different species of 5th instar silkworm larvae, their cocoons, and the fibers obtained from the silk cocoons. (**B**) The heavy chain (i.e., the N-terminus, β-sheets, amorphous loops, and the C-terminus) and the light chain, which is linked via disulphide bonds. Reprinted with permission from Ref. [45]. Copyright 2019 MDPI.

Silk proteins are produced by various insects such as silkworms, spiders, lacewings, glowworms, flies, and mites, and silks originating from silkworms and spiders are the most commonly used for biological applications. The most commonly used silk originates from *Bombyx mori*, a mulberry-feeding silkworm that produces higher quality fibers than most Saturniidae (non-mulberry-feeding worms) [37]. It is worth mentioning here that SF produced from different silkworms has different physico-chemical and biological properties due to differences in structural composition (Figure 3A). The natural process of cocoon formation (or spinning) involves extrusion of a combination of core structural SF protein (70–80%) and glue-like silk sericin protein (20–30%) through silkworm spinnerets in the form of fibers (Figure 3B) [38]. The structural transformation of SF silk I (primarily with an α-helix and random coil conformation) to silk II (anti-parallel β-sheets) occurs during the spinning process, and this provides the remarkable mechanical properties of semi-crystalline SF structures in cocoons. Due to the change in conformation into β-sheets, the SF protein present in cocoons is water stable. The silk sericin protein wraps around the fibroin and is generally soluble and can be removed by a degumming process. SF consists of a heavy (H) chain (~390 kDa) and a light (L) chain (~26 kDa), which are linked together via disulphide bonds to form an H-L complex [39]—and the glycoprotein P25 (~25 kDa) that is hydrophobically linked to the H–L complex [40]. The H-chain, L-chain, and P25 are assembled in a molar ratio of 6:6:1 to form silkworm silk [41]. The amino acid composition of SF from *B. mori* consists mainly of Gly (43%), Ala (30%), and Ser (12%). The hydrophobic domains of the H-chain contain the repetitive hexapeptide sequence of Gly-Ala-Gly-Ala-Gly-Ser and repeats of Gly-Ala/Ser/Tyr dipeptides that can form stable anti-parallel β-sheet crystallites. The amino acid sequence of the L-chain is non-repetitive, so the L-chain is more hydrophilic and is relatively elastic [42,43,44].

SF fibers have outstanding mechanical properties, such as a large break strain (4–26%), tensile strength (300–740 MPa), and toughness (70–78 MJ·m^−3^) [46]. Many researchers have used SF as a scaffold for load-bearing TE applications, especially in musculoskeletal TE, due to the excellent mechanical properties of SF. SF’s chemical composition and structure makes it highly biocompatible [47]. Biocompatibility is a key factor for the implementation of successful scaffolds because it enables cells to adhere to the scaffold surface and then migrate into the scaffold structure where they can proliferate and differentiate. In addition, it is important for the scaffold to cause no or only negligible immune responses after implantation [48]. Regenerated SF has been shown to have blood compatibility in several in-vivo experiments since 1989 [49], and SF has been widely used as a suture material since its approval by the US Food and Drug Administration in 1993 [50]. In 1995, Minoura et al. conducted pioneering research and successfully attached and grew fibroblast cells on *B. mori* SF matrices [51]. More recently, SF has been used as a substitute for collagen in cell culture to guide bone regeneration in rat calvarial defects, and this has demonstrated that collagen membranes can be replaced by SF membranes [52]. In vitro and in vivo studies have shown that there is no significant macrophage response to SF membranes or fibers [53]. Based on these properties, different silk-based medical devices have been approved by the FDA such as the bioresorbable surgical mesh Seriscaffold and the SF-based ligament graft SeriACL^TM^ [54].

Biodegradability and bioresorbability are also important attributes for biomaterials used in TE because the scaffolds need to gradually be replaced with the patients’ own cells and ECM over the course of recovery [55]. As a protein, SF is susceptible to proteolytic degradation by various enzymes such as protease XIV, alpha-chymotrypsin, and collagenase IA [56]. Enzymatically degraded SF polymers do not cause an immunogenic response [47], but because amyloid β-fibrils are associated with Alzheimer’s disease, there have been concerns about the risks associated with the degradation of the SF β-sheet structure. However, SF degradation products showed no significant cytotoxicity to neuronal cells in in vitro assays [57]. The SF degradation process is associated with the content of water-insoluble silk II structures (β-sheets), which are induced, for example, during methanol treatment or stretching, and water-soluble silk I structures (α-helices). With increasing amounts of silk II, and therefore increasing numbers of β-sheet structures, the degradation time increases, and thus the degradation rate of SF is highly dependent on the amount of β-sheet secondary structure present. Thus, the preparation of water-stable films from regenerated silk fibroin (RSF) solutions can address the need for rapidly degradable silk biomaterials [58], and these are in contrast to RSF films that are obtained via a slow air-drying process and that possess a reduced number of β-sheet structures and thus have faster degradation rates [59]. In addition, γ-radiation has also been shown to promote SF fiber degradation through the conversion of silk II to silk I [60].

## 3. SF-Based Morphologies

Both naturally derived and bioengineered silk proteins can be processed into various morphologies (Figure 1), including films, mats, artificial fibers, sponges, and hydrogels, and can even be used in 3D bioprinting.

### 3.1. Films

**Figure 4:**
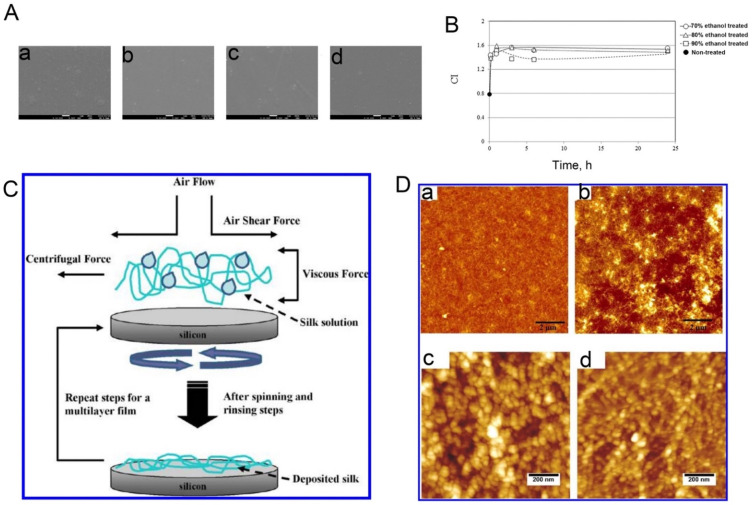
(**A**) Field emission scanning electron microscope images of spin-coated silk films and electrospun silk fibers: (a), the as-spun coated film; (b–d), the film treated in a 70%, 80%, and 90% ethanol solution, respectively. (**B**) Changes in the crystallinity index (CI) as a function of ethanol treatment time. Here, the CI is the ratio between the peak intensities at 1626 cm^−1^ and 1650 cm^−1^ in the infrared spectrum. Reprinted with permission from ref. [61]. Copyright 2016 Elsevier. (**C**) Schematic representation of the film assembly process according to vertical deposition method. (**D**) Topographical atomic force microscope images at different scales of silk monolayer films (a,c) and silk multilayer films (b,d). Reprinted with permission from ref. [64]. Copyright 2012 American Chemical Society.

Spin coating and vertical deposition are the main techniques used to fabricate RSF films. In the case of spin coating, an RSF solution and methanol are alternately coated onto glass substrates. As mentioned above, methanol is able to convert the structure of RSF from a high content of α-helices (Silk I) into a primarily β-sheet conformation (Silk II), and the concentration of alcohol affects the surface properties of the film. When treated with <80% ethanol, the fibroin film becomes jelly-like with a hydrated hydrogel as its outermost surface layer, while a fibroin film treated with >90% ethanol has a harder surface [61]. Another study showed that different methanol treatments can influence the surface properties that make the most important contribution to the success of a biomaterial implant, namely surface roughness, hydrophilicity, and rigidity [62] (Figure 4A,B). Vertical deposition is another method to prepare SF films and is considered a microfluidic process because it originates from the flow of the particle suspension into the deposits of particles already formed under the driving force of solvent evaporation [63,64] (Figure 4C,D). SF films have been extensively studied in biomedical applications such as drug delivery [65] and implantable devices thanks to their remarkable mechanical properties, their biodegradability, and their ability to support the adhesion, proliferation, and differentiation of different cell types in vitro [66].

### 3.2. Hydrogels

**Figure 5:**
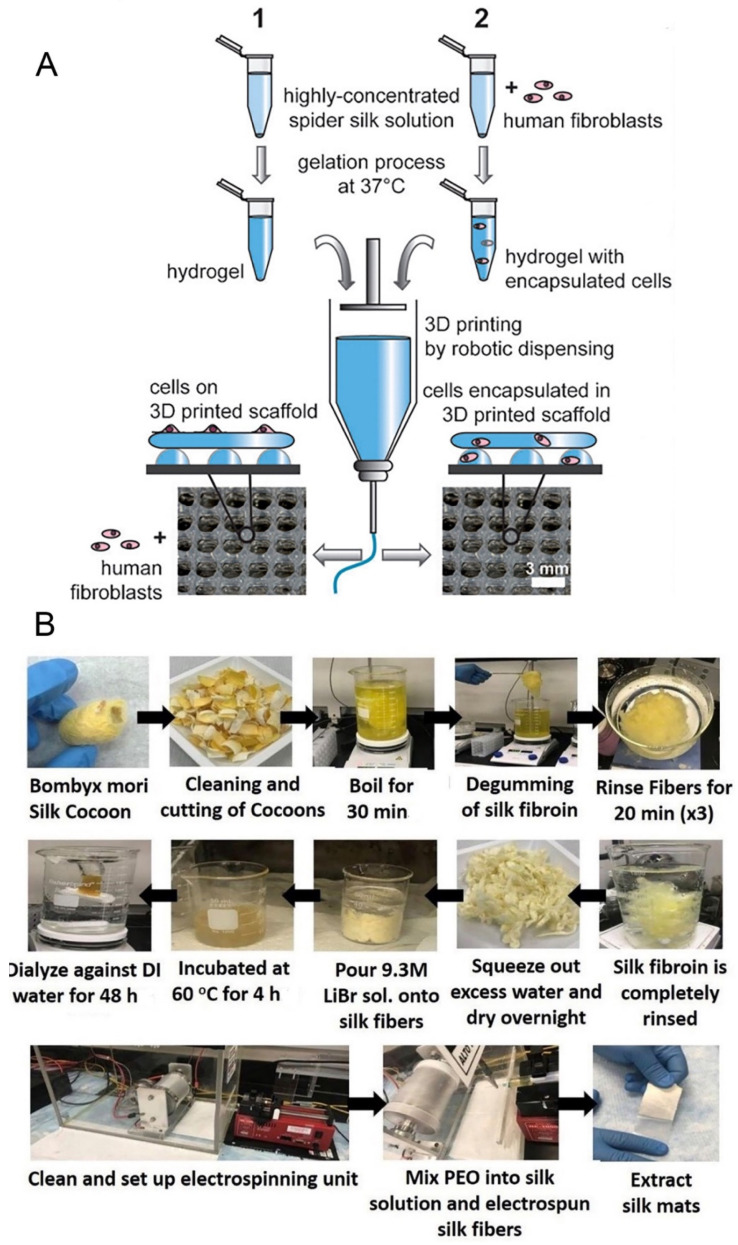
(**A**) 3D printing of spider silk hydrogel scaffolds by robotic dispensing. Reprinted with permission from ref. [75]. Copyright 2015 John Wiley and Sons. (**B**) Schematic representation of the SF preparation from raw silk cocoons through degumming process and solubilization. Electrospinning of SF solution leads to the formation of nanofibrous SF mats. Reprinted with permission from ref. [79]. Copyright 2020 American Chemical Society.

Hydrogels are water-swollen 3D polymer networks that provide multiple options for the delivery of cells and cytokines in TE. They are especially favorable for clinical applications because they offer the advantage of being injectable and thus are useful for cell seeding and encapsulation in the development of TE applications [67,68,69]. For example, Wang et al. encapsulated human mesenchymal stem cells into sonication-induced RSF hydrogels and reported the proliferation and viability of the cells in static culture after a week of in vitro cultivation [70]. The hydrogelation of SF is induced in aqueous SF solutions through high temperatures, low pH, high ionic strength, vortexing, sonication, freeze gelation, and electrogelation [71,72,73]. During the gelation process, structural changes in the SF occur from a disordered state to a β-sheet conformation that physically cross-links and stabilizes the gel [74]. The 3D hydrogel structure of recombinant spider silk proteins was produced through chain entanglement, then printed at room temperature using dispense plotting. Such spider silk scaffold can support cells to adhere and proliferate with good viability over at least one week [75] (Figure 5A). However, due to some unfavorable physical properties such as low viscosity, hydrogels made of *B. mori* SF are less suitable for bioprinting applications without additives. Here, strategies to blend the material with other (bio)polymers to enhance printability have been applied. For example, Chameettachal et al. successfully bio-printed SF-gelatin blends using dispense plotting at room temperature [76]. Moreover, supercritical CO_2_ technology can also form biomimetic porous SF scaffolds and can overcome the difficulty in obtaining complete solvent elimination and the preservation of the scaffold morphology at nanoscale limits thanks to the near zero surface tension of the supercritical CO_2_ plus solvent mixture and the very high diffusion coefficient of the supercritical system that allows complete solvent elimination from the sample [77]. Additionally, the porous SF scaffolds possess an ECM-like nanofibrous and interconnected porous structure, and the porosity and mechanical properties of the SF scaffolds can be controlled by the supercritical CO_2_ technology [78].

### 3.3. Mats

RSF mats or artificial silk fibers are commonly produced by fiber spinning techniques such as electro-spinning, wet-spinning, and dry-spinning. The electro-spinning technique can be employed to make polymeric nanofibrous scaffolds, and these can imitate the natural fibers of the ECM more closely in TE applications (Figure 5B). The technique is continuously evolving in order to gain more control over the process and its outcomes. RSF mats with porous structures can be fabricated on a large scale through electro-spinning, which is of great benefit for cell seeding in TE [80,81]. Traditionally, *B. mori* fibroin is dissolved in organic solvents such as hexafluoro-2-propanol (HFIP), hexafluoroacetone, or formic acid and spun by applying voltages between 2 kV and 30 kV. Non-woven mats containing fibers with diameters in the low nanometer range up to one micrometer have been generated with this set-up [82,83]. Recently, Yin et al. developed a finite element model that described the mechanical response of RSF–PCL mats under biaxial tension, and this model could be used to guide the design of RSF–PCL mats for TE applications [84]. Wet-spinning can also be used to fabricate RSF fibers, but only with fibers on the micrometer scale in contrast to the nanofibers produced from electrospinning. Wet-spinning allows the tuning of fiber morphologies and properties and allows for the combination with other biomolecules during the fabrication process [85]. For example, Chen et al. reported that polydopamine can modify the mechanical properties of RSF by affecting its hierarchical structure during the wet-spinning process [86]; however, producing large-sized proteins usually results in low production levels and high costs, and a complicated spinning process is not conducive to large-scale production. However, another study demonstrated that relatively small-sized recombinant spider silk can be used to spin high-quality fibers with a simple wet-spinning process [87]. In contrast to the former methods, dry-spinning does not require the use of organic solvents or coagulation baths and thus is more environmentally friendly. A newly developed approach uses centrifugal electrospinning to spin RSF nanofibers with increased structural and thermal stability compared to those obtained from standard electrospinning [88]. Moreover, compared to electrospinning, centrifugal electrospinning allows for a higher production rate at lower cost and is able to quickly produce highly interconnected non-woven nanofiber meshes [89].

### 3.4. Sponges

**Figure 6:**
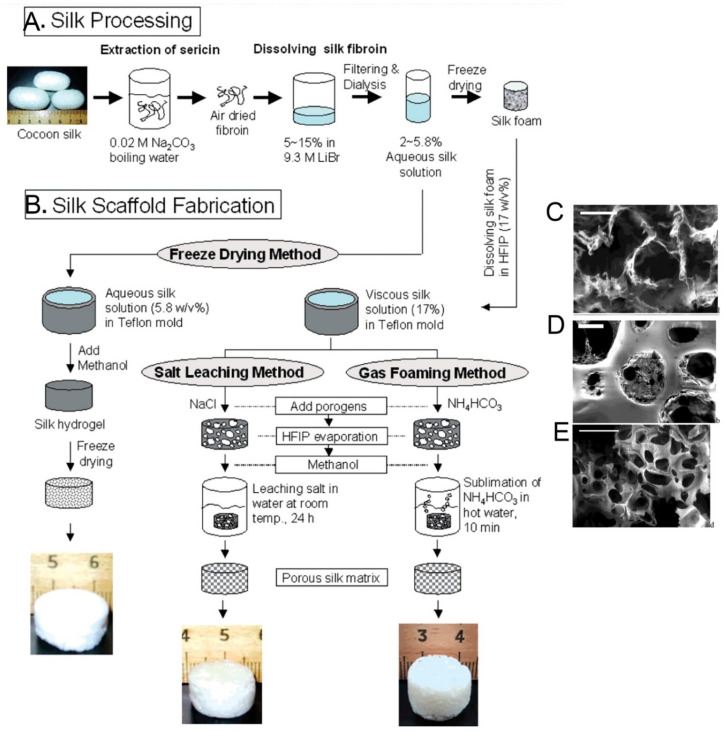
(**A**) Silk processing and (**B**) silk sponge scaffold fabrication flowchart. Scanning electron microscope images of the outer structure silk sponge scaffold formed by freeze-drying (**C**), salt leaching (**D**), and gas foaming (**E**) methods after methanol treatment. Reprinted with permission from ref. [92]. copyright 2004 American Chemical Society.

Sponges are made up of interconnected porous structures that closely mimic physiological environments in vivo. Their 3D porous structure allows for cell attachment, proliferation, and migration and facilitates nutrient and waste transport either by diffusion in a static environment or through perfusion, for example, in bioreactor setups. The pores can be formed with different sizes using salt leaching (porogens), gas foaming, or freeze-drying techniques [90] (Figure 6A,B). Processing parameters can be varied by using different solvents for the regeneration of SF, including organic solvents such as HFIP or aqueous solutions. HFIP-SF scaffolds are relatively resistant towards degradation and may take up to two years to degrade in vivo [91]. In a typical process the silk solution is dissolved in HFIP, and ammonium bicarbonate or sodium chloride particulates are added as a porogen, and the mixture is applied to Teflon disk-shaped molds. After scaffold formation, the salt is allowed to leach out of the construct in order to avoid negative influences on the cells grown on the scaffolds. The porous silk scaffolds are then air-dried and placed in a vacuum-dryer for 24 h [92]. This method leads to RSF sponge scaffolds with a highly homogeneous uniform pore size distribution as long as the sodium chloride particles have a homogenous size distribution (Figure 6D) [90]. Another method of regulating the pore size of sponges is via freeze drying; here, the freeze drying temperature, fibroin concentration, and pH of the RSF solution affect the pore size (Figure 6C) [93]. Gas foaming techniques can also form RSF sponges. For example, ammonium bicarbonate added to fibroin solutions will sublimate in hot water, thus aiding in the formation of porous sponge structures (Figure 6E) [92].

### 3.5. Micro-Patterning Structures

**Figure 7:**
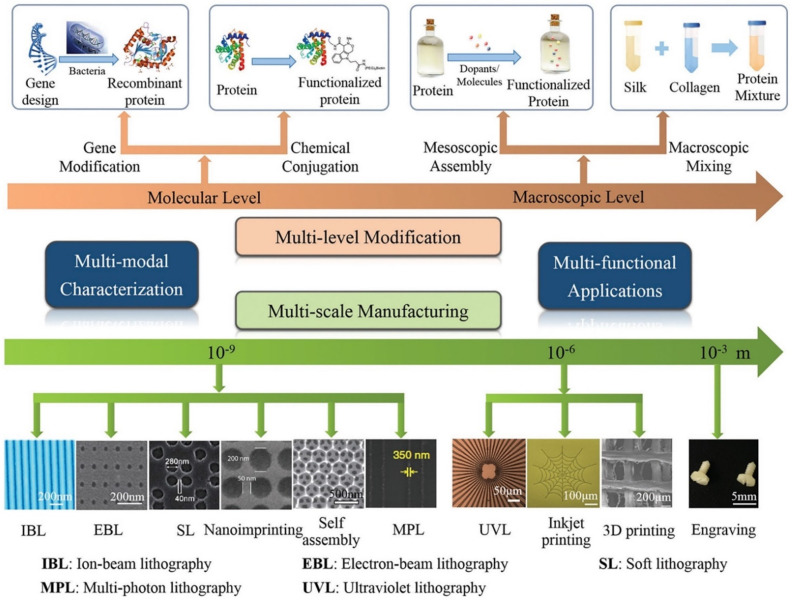
Schematic representation of the strategies used to modify, manufacture, and characterize silk materials. Reprinted with permission from ref. [97]. copyright 2018 John Wiley and Sons.

The ECM consists of complex micro- and nano-scale topologies that influence cellular behavior. Therefore, it is important to try to simulate as many of these topologies as possible in order to ensure that cells behave properly both in vitro and in vivo. The micro-patterning structure of RSF has been shown to affect cell migration, proliferation, and adhesion [94,95], and micro-patterning technology, which has attracted significant attention in recent years, enables the spatial control of cell behavior [96]. Lithography, including ultraviolet lithography (UVL), soft lithography (SL), scanning probe lithography (SPL), and electron-beam lithography (EBL), is a traditional technology for micro-patterning proteins onto substrates (Figure 7) [14,97]. Compared to UVL and EBL, SL is a convenient, inexpensive, and rapid technique, and SL offers the ability to control the molecular structures of surfaces and to pattern complex molecular arrangements that are relevant to biological application, i.e., to fabricate channel structures appropriate for microfluidics, and to pattern and manipulate cells [98]. UVL is performed by spin-coating RSF as a positive photoresist onto a silica substrate irradiated by an argon fluoride excimer laser through a patterned chromium mask. After washing the exposed areas with deionized water, the patterned RSF films show diffracted colors with a minimum line width of 1 µm. This process is water-based and does not require photoinitiators [99]. While SL requires fewer steps [98], it needs to be performed in a high-standard clean room and samples can be easily contaminated during fabrication [100]. In contrast, inkjet printing is a cost effective and flexible micro-patterning technique that is capable of patterning complex geometries with high precision [97]. Moreover, because inkjet printing is a non-contact technique, cross contamination of the final product is significantly reduced. Inkjet printing has been used as a micro-patterning technique to pattern self-assembled peptide nanofibers on RSF surfaces as a way to guide cell growth [101].

### 3.6. 3D Bioprinting Structures

**Figure 8:**
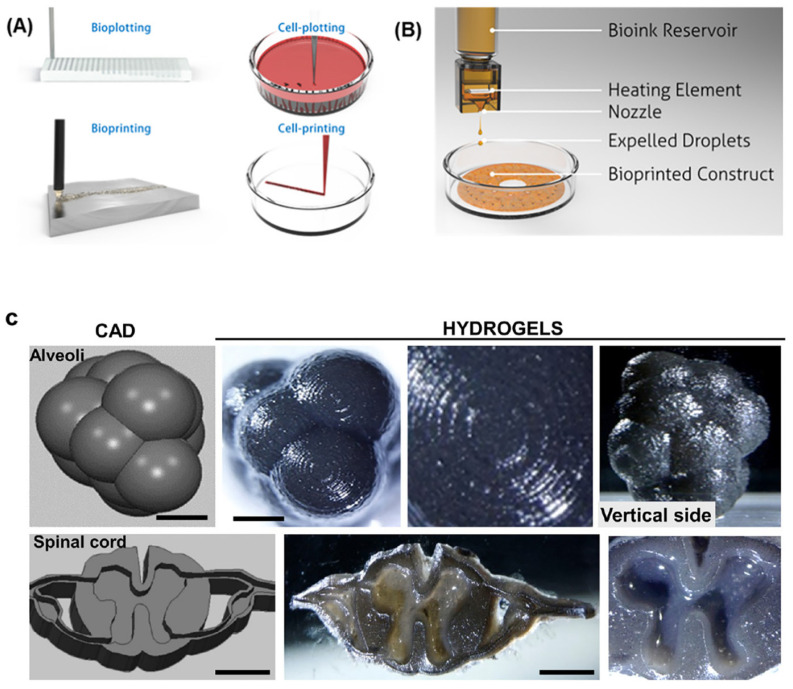
(**A**) Illustrations emphasizing the differences between bioplotting and bioprinting strategies. (**B**) Schematic of a bioinkjet style printing strategy. Reprinted with permission from ref. [102]. copyright 2016 American Chemical Society. (**C**) Representative sets of 3D DLP projections (CAD) and their corresponding printed specimens of alveolar structure and images of a transverse section of a human spinal cord. Reprinted with permission from ref. [105]. copyright 2020 American Chemical Society.

Bioprinting is a bottom-up additive manufacturing technology that can be used to manufacture complex and high-definition structures using computer-aided design tools. 3D bioprinting holds added advantages over conventional TE because it can integrate multiple architectural, biochemical, and mechanical cues by spatially and temporally tuning cell–cell junction stability, cell body shape, and cell alignment by dictating the cell–cell cohesiveness necessary to create complex and functional 3D tissue structures (Figure 8A,B). Biological inks (bioinks) are core to 3D bioprinting technology because these materials determine the structure and function of the tissues that are formed by providing stable biocompatible scaffolds in which cells can proliferate and differentiate [102]. Although 3D bioprinting has been applied in TE, there are still many challenges to overcome, including a limited range of materials and choices of cell type [103]. RSF is a unique material for 3D printing owing to its biocompatibility and polymorphic nature, and RSF can be printed via inkjet printing to fabricate “nest” shapes. Generally, 3D-printed RSF scaffolds are macroscopic in structure but can be regulated to form mesostructures and nanostructures by applying different mechanical stresses and using different dopants. For example, Sommer et al. [104] showed that the pore size of an RSF structure could be regulated by adding sacrificial monodisperse organic microparticles with varying sizes into RSF-based inks to create well-defined porous RSF scaffold structures. Recently, Kim et al. [90] showed that RSF can be chemically modified with glycidyl methacrylate to form a printable bioink (silk methacrylate) that can be printed to form complex structures, such as those in the brain and ear, using a digital light-processing 3D printer. The resulting 3D scaffolds possess strong mechanical properties that can be used in cartilage TE applications. Following this work, Ajiteru et al. [105] further improved the properties of the silk methacrylate bioink by conjugating it with reduced graphene oxide to form a composite bioink with better thermal stability and greater solubility (Figure 8C).

## 4. Application of RSF in TE

### 4.1. Bone Tissue Regeneration

**Figure 9:**
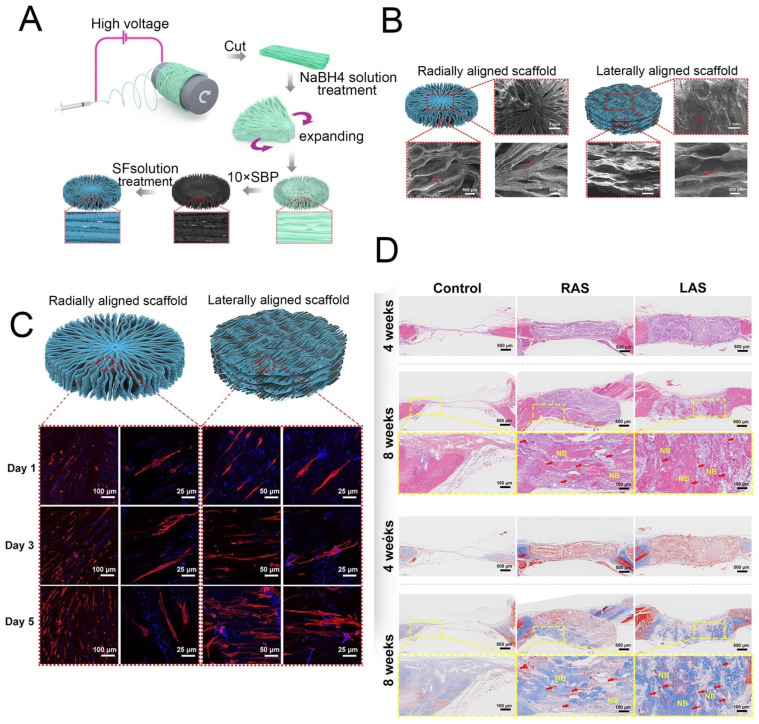
(**A**) Schematic showing the process used to fabricate the 3D radially aligned nanofiber scaffolds. (**B**) Top and side scanning electron microscope images of the radially aligned scaffold. (**C**) BMSCs proliferated on the radially aligned scaffolds and linearly aligned scaffolds. (**D**) Histological analysis of the scaffolds after implantation for 4 and 8 weeks. Reprinted with permission from ref. [26]. copyright 2021 IPC Science and Technology.

Bone is a hierarchically structured composite material composed of 35% organic parts and 60% inorganic matrix that, in addition to its obvious biological value, has been well studied by the materials engineering community because of its unique structural and mechanical properties. Bone is mainly composed of cells, fibers, collagen, and hydroxyapatite [106,107], and thus the scaffolding material for bone tissues must ensure matrix toughness and matrix deposition. In this context, SF is a rational choice due its high toughness and mechanical strength along with its good biocompatibility. SF-based bone TE is one of the most extensively studied TE approaches to date. Porous fibroin scaffold-based bone constructs are able to stimulate advanced development of bone tissues within 5 weeks when engineered within a bioreactor [24]. SF scaffolds also promote human mesenchymal stem cell-based healing of femoral defects in nude mice. Furthermore, the loading of human mesenchymal stem cells within an SF polyethylene oxide nano-fibrous composite scaffold with bone morphogenic protein-2 leads to the regeneration of bone-like tissue [108,109]. Incorporation of hydroxyapatite nanoparticles into silk matrix has been shown to improve bone regeneration in animals [110], and the incorporation of n-Hap within the fibroin sheet and subsequent culture of rat bone marrow mesenchymal stem cells showed the successful osteogenic differentiation of the cells [111]. These composite scaffolds can completely heal segmental bone defects in Sprague–Dawley rats after 12 weeks post-implantation. SF also guides full-thickness growth of calvarial defects (8 mm diameters) in rats within 12 weeks of implantation, and similar results are obtained when silk hydrogels or non-woven mats are used. Furthermore, one study showed that combinations of SF scaffolds with graphene could be an appropriate scaffold for bone tissue engineering [25]. A 3D polycaprolactone nanofiber scaffold modified by biomineralization and SF coating was explored according to electrospinning process (Figure 9A,B) and was used to elucidate the effect of the scaffold structure on bone regeneration. The results show that the scaffolds could guide cell arrangements and that the radially aligned scaffold demonstrated a greater ability to promote cell proliferation in vitro (Figure 9C) and could guide tissue arrangement and remodeling and support the significantly faster regeneration rate of bone tissue in vivo (Figure 9D) [26].

The vascularization of in-vitro tissue models is always a problem, and the pre-incubation of SF matrices with cells prior to implantation in animals increases the vascularization in neo-implants [112]. Under such conditions, the rate of vascularization is directly proportional to the in-vitro incubation time. Co-culture of osteoblasts with endothelial cells on SF scaffolds results in the formation of micro-capillary and pre-vascular structures, and upon implantation, these premature micro-capillaries survive the host defense system and become functional micro-capillaries [113]. Further investigations on scaffold architectures and pore distribution are needed to obtain completely vascularized 3D bone tissue.

### 4.2. Skeletal Muscle TE

Muscle is a contractile tissue mainly found in three types, namely skeletal, cardiac, and smooth muscles [114]. Skeletal muscle is composed of longitudinally arranged myofilaments and refers to tissues that transform the chemical energy stored in glucose into mechanical force to either provide locomotion of the organism itself or the movement of internal organs. Hence, skeletal muscle is one of the most crucial functional tissues of the body, and there are more than 600 distinct skeletal muscles in the human body [115]. Traumatic injury or diseases such as myopathies and amyotrophy can severely impair their functions, and various surgical transplantation and transposition techniques have been developed to make up for the loss of muscle and to restore their functions [114]. However, such procedures have had limited success, and therefore muscle TE is regarded as an alternative strategy to ameliorate this situation. Despite many efforts and even though the engineering of certain human organs has recently entered clinical practice, skeletal muscle tissue regeneration is still a technological challenge [114]. Recently, Liang et al. developed an electro-spun nanofibrous scaffold composed of PLA/SF/collagen and showed that this bio-composite is non-cytotoxic. Moreover, the blended scaffold led to the increased growth of spindle-shaped and regularly arranged myoblasts [116]. Similarly, Shen et al. prepared a poly(ester-urethane) scaffold whose surface was modified by SF in order to improve the scaffold’s performances both in vitro and in vivo [111]. Compared with unmodified scaffolds, skeletal muscle cells showed better proliferation and differentiation on modified scaffolds with enhanced hydrophilicity and better biocompatibility. In addition, they showed that SF-bonded poly(ester-urethane) scaffolds are more compatible with the surrounding tissue and are degraded faster than the non-SF-modified group when implanted subcutaneously in rats.

### 4.3. Cartilage Tissue Regeneration

Cartilage is an avascular and non-innervated connective tissue that protects the subchondral bones from high stresses in the joints and plays a crucial role in maintaining locomotion of the body. Unfortunately, cartilage undergoes progressive degeneration due to its limited self-repair ability after injury or damage. Currently, many surgical procedures have been used to restore cartilage, but mostly with unsatisfactory outcomes. Therefore, TE provides a hopeful approach for cartilage repair and replacement.

**Figure 10:**
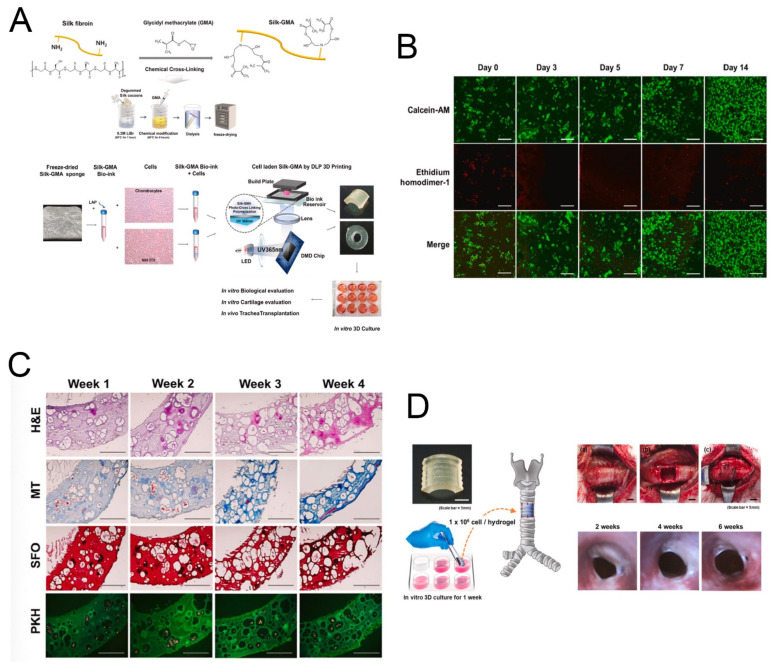
(**A**) Schematic presentation of methacrylation of SF with GMA (Silk-GMA) and bio-printing of chondrocytes with Silk-GMA by a digital light processing 3D printer. (**B**) Confocal microscopic images for the Live/Dead assay with Calcein-AM (live cells, green fluorescence) and ethidium homodimer-1 (dead cells, red fluorescence) staining showing that human chondrocytes proliferated well in 30% Silk-GMA hydrogel for up to 2 weeks of cultivation. (**C**) In vitro histological detection of human chondrocyte-laden Silk-GMA hydrogel for cartilage tissue formation. (**D**) Schematic summaries of chondrocyte-laden Silk-GMA hydrogel transplantation and endoscopic observation of rabbit trachea for 6 weeks after transplantation. Reprinted with permission from ref. [30]. copyright 2020 Elsevier.

SF scaffolds can be fabricated into different morphologies to enhance the production of cartilaginous ECM. For example, Cai et al. prepared an SF-coated PLA film system and found that the hydrophilicity of the PLA film was improved by the silk coating, and this reinforced the attachment and proliferation of rat osteoblasts [29]. A recent study has demonstrated that SF—as a natural polymer fabricated with glycidyl-methacrylate (Silk-GMA) gel—can be used for digital lighting processing (DLP) 3D printing (Figure 10A) [30]. The Silk-GMA gel exhibited great cell biocompatibility in vitro (Figure 10B,C) and provided an excellent environment for growth and maintenance of chondrocytes. In addition, the Silk-GMA hydrogel replaced the defective part of the trachea and could serve as a guide for the regeneration of tracheal tissues (Figure 10D). Growth factors can accelerate the process of tissue (re)generation, and therefore scaffolds with appropriate growth factors can provide a biologically active substrate for tissue formation and are considered as an alternative to autologous or allogenous cartilage implants [117]. For example, insulin-like growth factor is a regulatory molecule involved in chondrogenesis, and it can be incorporated within scaffolds for better chondrogenic outcomes. In addition to growth factors, bioreactors provide mechanical stimulation for the proper maturation of cartilaginous constructs [31,117]. Other factors to be taken into consideration for regenerating cartilaginous tissues are cell sources [118], scaffold architecture, pore size, and pore distribution. Taken together, these studies have shown that the incorporation of bioactive growth factors into SF scaffolds along with appropriate scaffold architecture can produce better chondrogenic outcomes.

In addition, osteochondral (OC) tissue consists of articular cartilage and subchondral bone that are present in human joints and are connected by a stable interface that integrates these two elements into a single complex tissue [119]. OC defects or damage can happen in any joint in the human body and can affect the articular cartilage, the underlying subchondral bone, or the interface between the two tissues [119]. Considering the heterogeneity of OC tissue, innovative biomaterial structures containing different mechanical and biological properties according to the target OC tissue layers are needed. Considering the unique properties of SF for biomedical applications as described above, the fabrication of useful SF-based scaffolds has been extensively investigated with very positive results in the repair and regeneration of OC tissues. Saha et al. [120] evaluated the osteocyte and chondrocyte-inducing ability of acellular mulberry and nonmulberry SF scaffolds as an implantable platform in OC therapeutics. Zhang et al. [121] developed a bioink consisting of decellularized ECM and SF to print the bilayered scaffold. Each layer of the scaffold had suitable mechanical strengths and degradation rates, and the scaffolds loaded with growth factors promoted osteochondral regeneration in a rabbit knee joint model. SF lacks a bioactive domain for cell adhesion, proliferation, and differentiation, thus limiting its therapeutic efficacy. However, Chen et al. [122] engineered an elastin-like polypeptide (Val-Pro-Gly-Xaa-Gly) to modify SF fibers via simple and non-toxic dehydrothermal treatment, and they found that this scaffold could enhance mature bone and cartilage tissue formation compared to the naked SF scaffolds. Furthermore, the novel design of an integral bilayer scaffold combined with a photocurable silk sealant for osteochondral repair has been reported [32].

### 4.4. Periodontal Tissue Regeneration

The state of the art for the regeneration of mouth tissues is placing implants at the defect location and encouraging them to integrate into the implantation site. However, it is important that the tooth socket remain for implants, and this is more related to bone regeneration than regenerating the teeth themselves. The first approaches in this field of application were realized using *B. mori* SF-based scaffolds prepared via freeze-drying, and these were studied in attempts to preserve the jaw ridge [33]. An appropriate rate of material resorption was found for SF scaffolds with pore sizes around 200 nm and nano-hydroxyapatite reinforcements, which were additionally mineralized in vitro. These scaffolds showed osteogenic differentiation in pre-osteoblast MC3T3 E1 cells after 21 days, and the interaction with human bone marrow stromal cells showed good biocompatibility [33]. Another study focused on culturing stem cells from human exfoliated deciduous teeth on sponges prepared from silkworm cocoon cuts. Cell proliferation on SF scaffolds has been confirmed, but scaffolds for endodontic repair, which can simulate dynamic dental pulp repair, are only beginning to be used in the field [123].

### 4.5. Vascular Tissue Regeneration

**Figure 11:**
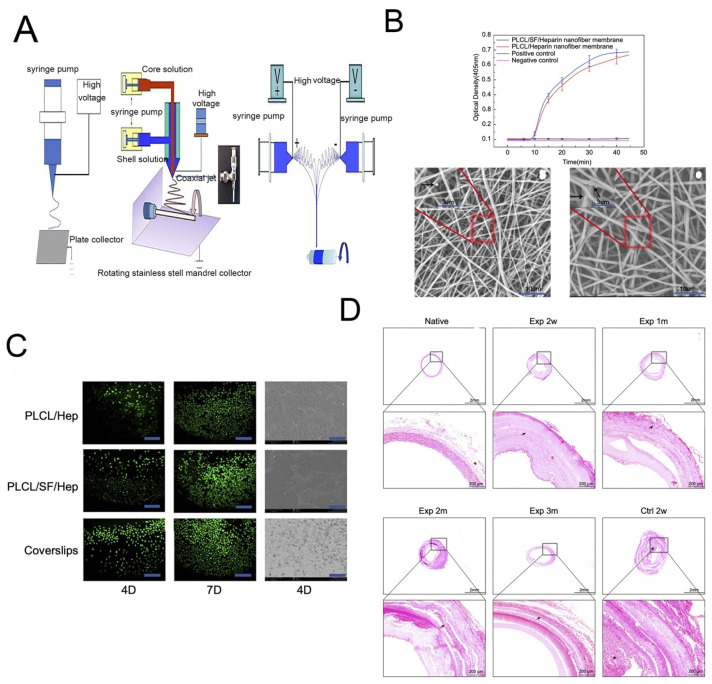
(**A**) The schematic diagram of different electrospinning devices. (**B**) The ability to resist thrombosis. (**C**) Live cell staining photomicrograph of HUVECs and scanning electron microscope images of HUVECs grown on different materials at day 4. (**D**) The HE stains of different vascular scaffolds. Reprinted with permission from [128]. copyright 2019 Dove.

SF-based regenerated vascular tissues are clinically used as flow diverting devices and stents [124]. In the case of a study related to flow-diverting devices, two out of three patients showed promising outcomes, suggesting that silk is an attractive option for treating fragile blood blister-like aneurysms. SF stents are also employed in the reconstruction of arteries after intra-cranial aneurysms. A tubular ~3 mm blood vessel with a thickness of 0.15 mm was fabricated from silk, and the average tensile strength was 2.42 MPa [125]. The burst strength of silk tubular vessels is 811 mm Hg compared to 1800 mm Hg for the gold standard saphenous veins [126]. The implantation of vascular grafts of SF composites from *B. mori* and from transgenic silkworms into the rat abdominal aorta resulted excellent patency (ca. 85%) after a year [127]. Composites of SF and human-like collagen or double-raschel knitted SF-poly(ethylene glycol diglycoldiglycidyl ether) have been successfully used to develop vascular constructs. While the former composition is able to provide good tensile strength to the construct [34], the latter is able to avoid early thrombosis. Jin et al. evaluated the remodeling capabilities of poly(L-lactide-co-ε-caprolactone) (PLCL), SF, and heparin (Hep) bi-layered scaffolds and PLCL/Hep bi-layered scaffolds by in vivo transplantation (Figure 11A) [128]. The results showed that the number of adherent platelets on PLCL/SF/Hep nanofiber membranes was much smaller than on the PLCL/Hep nanofiber membrane, indicating that the addition of SF can greatly improve the blood compatibility and increase its antithrombotic capacity (Figure 11B). Moreover, the addition of SF greatly improved the biocompatibility of the materials according to calcein staining after the endothelial cells were cultured on the different materials (Figure 11C). The H&E staining confirmed that the PLCL/SF/Hep scaffolds retained patency and no thrombus formed within 3 months of surgery, which was similar to what was seen when using native rabbit carotid artery (Figure 11D). An aqueous gel-spinning process has also been employed to design a blood vessel-like tubular structure [35]. Critical requirements for designing blood vessels include survival under changes in blood pressure, the ability to sustain cyclic loading, compatibility with the adjacent host vessels, and an anti-thrombotic lining [129]. SF possesses an anti-thrombotic surface with good resistance to high shear stress and blood flow pressure [130]. Moreover, SF fibers also support the intercellular contact of endothelial cells [36]. However, the challenge of including a selection of suitable cell sources remains. For example, primary human endothelial cells seeded onto silk fiber are unable to fill the gaps between two adjacent silk fibers. The optimization of co-culture of endothelial cells and smooth muscle cells to obtain a well-organized endothelium, the sulfation of silk for better anti-coagulant activity, and coating with Matrigel to enhance endothelium coverage are suggested developments for the construction of native vascular tissue.

## 5. Future Prospects

Tissue regeneration for therapeutic purposes is target specific, and to complement the functionality of living systems, the constructed tissue must successfully avoid the body’s immune system. SF-based designs allow easy control of matrix morphology, degradation rate, and conformational adhesion to underlying tissues with low immunotoxicity and good biocompatibility. Recent advancements in our understanding of silk structure and processing have opened up new opportunities for the use of various forms of silk in tissue regeneration. Silk systems will be particularly useful for applications where slow biodegradation and good mechanical properties are required, such as for bone and skeletal muscle tissues. The successful application of silk-based materials in tissue engineering depends on furthering our understanding of their long-term biocompatibility, their biodegradability, and the effects of their degradation products along with how to tune silk morphologies for tissue-specific requirements. Furthermore, the use of hybrid materials that incorporate silk into different matrix morphologies shows promise in this regard.

## Figures and Tables

**Table 1:** Overview of silk fibroin-based tissue engineering application.

Silk Source	Material	Morphology	Cell Types	Biocompatibility Study	Target Tissue
*Bombyx mori* silk fibroin [24]	SF	Porous and three-dimensional (3D) silk scaffold	Human mesenchymal stem cells (hMSCs)	In vitro and in vivo	Bone
*Bombyx mori* silk fibroin [25]	Graphene/SF	3D porous Graphene/SF Scaffolds	Rat bone marrow mesenchymal stem cells (rBMSCs)	In vitro	Bone
*Bombyx mori* silk fibroin [26]	Polycaprolactone/SF	3D polycaprolactone nanofiber scaffold	Bone marrow mesenchymal stem cells (BMSCs)	In vitro and in vivo	Bone
*Bombyx mori* silk fibroin [27]	SF/ceramic (calcium sulphate (CaSO_4_), beta tricalcium phosphate (β-TCP), beta tricalcium phosphate with hydroxyapatite (β-TCP-HA), and alkaline phosphatase (ALP))	RSF scaffolds	hBMSCs	In vitro	Bone
*Bombyx mori* silk fibroin [28]	Polyhydroxyalkanoates (PHAs)/SF	Electrospun poly(3-hydroxybutyrate-co-3-hydroxyhexanoate)/silk fibroin film	Human umbilical cord-derived mesenchymal stem cells (hUC-MSCs)	In vitro	Bone
*Bombyx mori* silk fibroin [29]	Poly (d,l-lactic acid) (PDLLA)/SF	Films	Osteoblasts	In vitro	Cartilage
*Bombyx mori* silk fibroin [30]	SF-glycidyl methacrylate (Silk-GMA)	Digital Light Processing (DLP) 3D printing Silk-GMA hydrogel	NIH/3T3 mouse fibroblast cells	In vitro and in vivo	Cartilage
*Bombyx mori* silk fibroin [31]	SF	Sponge	Chondrocyte	In vitro and in vivo	Cartilage
*Bombyx mori* silk cocoons [32]	SF/Methacrylated silk fibroin sealant (Sil-MA)	Bilayer silk scaffold and Sil-MA hydrogel	BMSCs	In vitro and in vivo	Osteochondral
*Bombyx mori* silkworm cocoons [33]	SF	Porous SF scaffold	Pre-osteoblasts (MC3T3-E1)	In vitro and in vivo	Tooth
*Bombyx mori* silkworm cocoons [34]	Poly(L-lactide-co-ε-caprolactone) (PLCL)/SF/heparin (Hep)	PLCL/SF/Hep nanofiber membrane	Human umbilical vein endothelial cells (HUVECs)	In vitro and in vivo	Vascular
*Bombyx mori* silkworm cocoons [35]	SF	Silk tube	Human coronary artery smooth muscle cells, HUVECs	In vitro and in vivo	Vascular
*Bombyx mori* silkworm cocoons [36]	SF	Silk fibroin nets	HUVECs	In vitro	Vascular

## Data Availability

Not applicable.
